# Evaluation of methylene diphenyl diisocyanate as an indoor air pollutant and biological assessment of methylene dianiline in the polyurethane factories

**DOI:** 10.4103/0019-5278.50723

**Published:** 2009-04

**Authors:** Mirtaghi Mirmohammadi, M. Hakimi Ibrahim, Anees Ahmad, Mohd Omar Abdul Kadir, M. Mohammadyan, S. B. Mirashrafi

**Affiliations:** School of Industrial Technology, University Science Malaysia, 11800, Pulau Pinang, Malaysia

**Keywords:** Air pollution, diisocyanates, polyurethane factories, sampling and analysis, statistical model

## Abstract

Today many raw materials used in factories may have a dangerous effect on the physiological system of workers. One of them, which is widely used in the polyurethane factories, is diisocyanates. These compounds are widely used in surface coatings, polyurethane foams, adhesives, resins, elastomers, binders, and sealants. Exposure to diisocyanates causes irritation to the skin, mucous membranes, eyes, and respiratory tract. Methylene dianiline (MDA) is a metabolite of methylene diphenyle diisocyanate (MDI), an excretory material of worker's urine who are exposed to MDI. Around 100 air samples were collected among five factories by the Midget Impinger, which contained DMSO absorbent as a solvent and Tryptamine as a reagent. Samples were analyzed by high-performance liquid chromatography with an EC\UV detector using the NIOSH 5522 method of sampling and analysis. Also, fifty urine samples were collected from workers by using William's biological analysis method. The concentration of MDI in all air samples was more than 88 μg/m^3^, showing a high concentration of the pollutant in the workplaces in comparison with the NIOSH standard, and all the worker's urine was contaminated by MDA. The correlation and regression tests were used to obtain statistical model for MDI and MDA that is useful for prediction of diisocyanates pollution situation in the polyurethane factories.

## INTRODUCTION

The lower molecular weight isocyanates tend to volatilize at room temperature, creating a vapor inhalation hazard. Conversely, the higher molecular weights isocyanates do not readily volatilize at ambient temperatures but are still an inhalation hazard if aerosolized or heated in the work environment. The feature common to all diisocyanates (monomers) is the presence of two -N=C=O (isocyanate) functional groups attached to an aromatic or aliphatic parent compound.[1] These compounds are widely used in surface coatings, polyurethane foams, adhesives, resins, elastomers, binders, and sealants. In general, the types of exposures encountered during the use of isocyanates (i.e., monomers, pre-polymers, polyisocyanates, and oligomers) in the workplace are related to the vapor pressures of the individual compounds.[2] Isocyanates exist in many different physical forms in the workplace. The workers are potentially exposed to the unreacted monomer, pre-polymer, polyisocyanate, and/or oligomer species found in a given product formulation.[3] They can also be exposed to partially reacted isocyanate-containing intermediates formed during polyurethane production.[4] The latter might be more hazardous as many reactions involving isocyanates are exothermic in nature and can provide the heat for their volatilization.[5] As the exposure limits decrease, the volatility of solid materials becomes an issue. To reduce the vapor hazards associated with the lower molecular weight diisocyanates, pre-polymer and polyisocyanate forms of these diisocyanates were developed and have replaced the monomers in many product formulations. An example is toluene diisocyanate (methylene diphenyle diisocyanate [MDI]), which consists of three molecules of MDI monomer joined together to form a higher molecular weight oligomer having similar characteristics to that found in the monomer. Many pre-polymer and polyisocyanate formulations contain a small fraction (usually less than 1%) of unreacted monomer.[6]

Exposure to isocyanates is irritating to the skin, mucous membranes, eyes, and respiratory tract. The most common adverse health outcome associated with isocyanate exposure is asthma due to sensitization. Less prevalent are contact dermatitis (both irritant and allergic forms) and hypersensitivity pneumonitis.[7] Contact dermatitis can result in symptoms such as rash, itching, hives, and swelling of the extremities. A worker suspected of having isocyanate-induced asthma/sensitization will exhibit the traditional symptoms of acute airway obstruction, e.g. coughing, wheezing, shortness of breath, tightness in the chest, and nocturnal awakening. An isocyanate-exposed worker may first develop an asthmatic condition (i.e., become sensitized) after a single (acute) exposure, but sensitization usually takes a few months to several years of exposure.[8]

## MATERIALS AND METHODS

**Figure 1:**
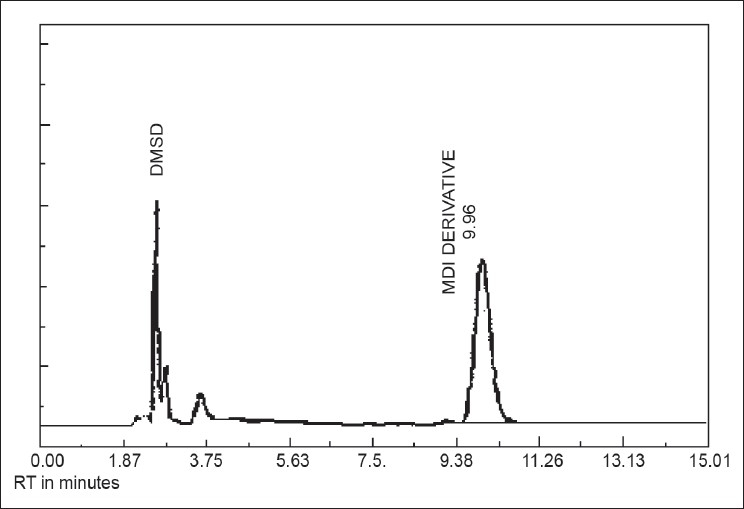
MDI chromatogram by HPLC

A total of five factories of Iran were selected, which have about 500 workers, the factories produce foaming or polyurethane foams and the workers are exposed to MDI through indoor air pollution. There were some workers who do not work fulltime in the workplace. They work as an officer and sometimes people coming and going into the workplace as unexposed workers were also included. The air sampling and analysis of isocyanates from indoor air are divided into four steps: collection, derivatization, sample preparation, and identification.[5] Samplers have been calibrated by a flam flow meter. The Midget Impinger SKC glass personal inhalable sampler (SKC, Houston, TX 77095-5027 USA), with a mini personal sampler pump SIBATA was used. All the samplers with the midget impinger connected to the mini personal sampler pump were fixed to work stations near the source of pollution. The air sample was collected in three working shifts for 2 h at a flow rate 2 L/min in an impinger containing a solution of reagent in DMSO in addition with tryptamine.[5] The air samples were collected at the every 2 h of the work shift. After passing 120 L of air, the entire sample medium was transferred to the laboratory for analysis. Sample handling and preparation were carried out to make it compatible with the analytical procedure as per standard methods. The first step in the analysis of a solution is derivatization of isocyanates for the separation through high-performance liquid chromatography (HPLC) for their qualitative as well as quantitative analysis [Figure 1].

The biological sampling was performed by collecting the workers' urine at the end of the working shift, collected into polystyrene containers with citric acid and transferred to the laboratory for analysis by GC Mass.[9,10]

### Calculations and statistics

The workers are divided individually into two groups: office personnel and factory workers. The first group consisted of 100 people and they had least exposures while the other group consisted of 400 persons who were sufficiently exposed to MDI. A total of 100 samples were collected randomly from working places inside the factory for the exposed persons by statistic sampling. However, only five samples were collected from offices as blank samples for least-exposure persons. All the air samples were analyzed in the laboratory for MDI using HPLC through the standard method of analysis[5] and, for biological samples, were analyzed using the GC Mass trough method of Williams.[10] Statistical analysis was performed using ANOVA test and regression through SPSS.

## RESULTS AND DISCUSSION

The analysis of the data shows a high concentration (>95 *μ*g/m^3^) of pollutant (MDI) in comparison with the NIOSH exposure limit value (50 *μ*g/m^3^). The result of the analysis of the air samples by HPLC has shown that the MDI concentration ranged from 95 to 101 *μ*g/m^3^. In Table 1, the maximum indoor proportional humidity in five factories was 45% (factories A and B), 43% (factories C, D, and E). The minimum indoor proportional humidity in all the five factories was 43% (factories A and B) and 43% (factories C and D and 41% for factory E). The overall mean of indoor proportional humidity was 43.7%.

**Table 1:** Maximum and minimum reading of indoor air variables in the five MDI polyurethane factories

Factories code Variables	A	B	C	D	E	N Stations	Total
Max MDI pollution concentration (*μ*g/m^3^)	101	98	97	96	95	300	101
Min	98	98	96	95	93		93
Mean	98.89	98	96.95	95.57	93.84	96.64	
Max Relative humidity (%)	45	43	43	43	300	45	
min	45	45	43	43	41		41
Mean	45	45	43	43	42.58	43.7	
Max Wet temperature (°C)	23	25	25	27	27	300	27
min	23	23	25	25	27		23
Mean	23	24.33	25	26.33	27	25.16	
Mean Dimension of factory (m^3^)	6300	8600	9800	11,200	12,500	5	9680
Mean Altitude (m)	1200	1200	1100	890	22	5	882

In Table 1, the maximum dry indoor temperature among the five factories was 23–27°C. The minimum wet indoor temperature among the five factories was 23–27°C. The overall mean dry indoor temperature was 25.16°C. The maximum wet indoor temperature among the five factories was 28–29°C. The minimum wet indoor temperature among the five factories was 28–29°C. The overall mean wet indoor temperature was 28.72°C. The mean area of the workplace for the five polyurethane factories (A, B, C, D, and E) was 6300, 8600, 99,800, 11,200, and 125,00 m^3^, respectively.

The relationship between multifactor scores and the MDI pollution were studied to understand the behavior of indoor air components with respect to different polyurethane factories. Regression analysis was used to assess the interactive behavior for MDI pollution and indoor air parameters. It is clear that all of the evaluated parameters associated with MDI pollution are well defined.

A general regression model were extracted from parameter analysis of indoor air pollution factor and MDI pollution because the α rate is less than 0.001 (α < 0.001). Therefore, this prediction model at the level of 0.001 is significant for all the numbered in the regression table have a strong relationship with MDI pollution concentration in the polyurethane factories, as it shown in Table 2.

**Table 2:** Coefficients of regression model for MDI pollutant and polyurethane indoor air parameters

Model		Unstandardized coefficients		Standardized coefficients		
			
		B	Std. error	Beta	t	Sig.
4	Constant	16.619	4.740		3.506	0.001
	Relative humidity (%)	1.238	0.090	0.652	13.757	0.0001
	Dimension factory (m^3^)	0.000	0.000	−0.096	−8.414	0.0001
	Dry bulb temperature (°C)	1.276	0.201	0.284	6.357	0.0001

One predictive regression model was obtained from Table 3 about the relationship between polyurethane indoor air parameters and the MDI pollution situation based on the predictive statistical modeling formula as follows:

**Table 3:** Relationship test between air sample results and urine sample results

Model		Sum of squares	df	Mean square	F	Sig.
1	Regression	912.73	1	912.73	46.737	0.0001(a)
	Residual	253.876	13	19.529		
	Total	1166.606	14		

a Predictors: constant, MDA standard pollution. b Dependent variable: MDI standard pollution.

Y = β_◊_ + β_1_ Rh + β_2_ D + β_3_ Td

Where:

Y = MDI concentration

β_◊_ = Model constant

β_1_ = Relative humidity coefficient

β_2_ = Dimension of factory coefficient

β_3_ = Dry bulb temperature coefficient

χ1 = Relative humidity (Rh)

χ2 = Dimension of workplace (D)

χ3 = Dry bulb temperature (Td)

The predictive regression modeling was designed for this study about indoor air pollution parameters and MDI pollutant concentration. With this data, the model is:

Y = 16.619 + 0.652 × Rh − 0.096 × D + 1.276 × Td

This model may help in assessing the psychometric parameters in the polyurethane factories and estimate the pollution situation of MDI in the polyurethane workplaces.

All the isocyanates, after inhalation by workers in the work places, are metabolized or broken down in the body and eliminated in the urine. The level of isocyanate metabolites in the urine is an indicator of how much isocyanate has been absorbed and how well the controls are working. The levels of MDA are reported as “*μ*mol/mol creatinine”. Creatinine is found in everyone's urine and can be used to adjust the level of MDA to compensate for dilute or concentrated urine. The guidance value for MDA is the level of 2, and each sample above the guidance value is contaminated.[11]

**Figure 2:**
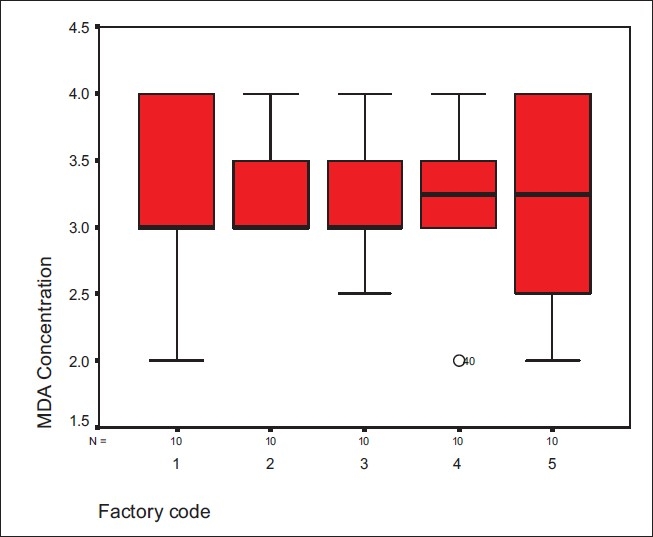
MDA concentrations in different workplace areas

The maximum concentration of the metabolite of MDI (methylene diphenyl diisocyanate), which was got from worker's urine in all of the MDI polyurethane factories was 4 *μ*mol/mol creatinine and the minimum MDA was 2 *μ*mol/mol creatinine. The means of the MDA were 3.5 and 3.15 *μ*mol/mol creatinine for factories 1 and 2, respectively, 3.4 *μ*mol/mol creatinine for factories 3 and 5, and 3.5 *μ*mol/mol creatinine for factory 4. All the MDI polyurethane workers urine results indicated high pollutant with MDI because the rate of MDA in their urine was 4 *μ*mol/mol creatinine, as shown in Figure 2.

The AVOVA statistical test was carried out for determining the significant relationship between MDI air samples and urine samples from workers.

Table 3 shows that there is a strong relationship between air sample results and urine sample results. It is implied that all urine samples having a high concentration of metabolite of MDI were relevant to a high concentration of air samples in the polyurethane factories. Also, it was inferred from the table that the statistical model for this group of data is significant and the linear relationship between two variables is significant (*P* < 0.001).

There is a strong correlation between MDI air sample results and urine sample results from workers, which means that all the data obtained from the urine of workers have a straight correlation with the data of air samples and with *P* < 0.001. It can be inferred that two different variables were correlated together.

The predictive statistical model for MDI pollution was inferred from Table 4. The obtained data from air analysis and urine analysis were interred for make a statistical modeling and the β-coefficients were used to make the following model:

**Table 4:** Regression model coefficient for MDI and MDA

Model		Unstandardized coefficients		Standardized coefficients	t	*P*-value	95% confidence interval for B	
						
		B	Std. error	Beta			Lower bound	Upper bound
1	Constant	42.485	6.423		6.615	0.0001	28.610	56.360
	MDA standard pollution	13.075	1.913	0.885	6.836	0.0001	8.943	17.207

a Dependent variable: MDI standard pollution.

Y = a + b X

Where:

Y is an independent variable (MDA)

a is the model constant

b is the model coefficient

X is the dependent variable (MAI)

Y = 42.485 + 13.075 X or MDA = 42.485 + 13.075 MDI

Coefficients of this model (a and b) in Table 4 were significant at the level of 0.001 (*P* < 0.001) for both constant coefficient and MDI coefficient, which means that it can be used for the prediction of MDA concentration in the polyurethane factories' indoor air from the rate of MDI concentration from polyurethane workers at a reliability level of 99.999%. It implies that when MDI concentration is determined from polyurethane air, it must be multiplied with the constant number 42.485 and product plural with the constant number 13.075, product of this equation predicting the concentration of MDA in the workers' urine.

The measurement of isocyanates in air is a challenging sampling and analytical problem for several reasons. Isocyanates can exist in air either as vapor or as aerosol, having a wide range of particle sizes. Isocyanates are very reactive and hence unstable and give many different chemical species, even in the same air sample, which need to be quantified. Pure analytical standards are not available for the vast majority of these isocyanate species and qualitative standards (bulk products) do not account for isocyanate species generated during polyurethane formation or breakdown. Finally, to measure isocyanates at levels corresponding to current monomer exposure limits, analytical methods must be very sensitive. Because of the complex problems associated with accurate sampling and analysis of total isocyanate group in air, existing methods have limitations. To assess these limitations and to make rational decisions in choosing methodologies or making improvements to existing methodologies, it is useful to break down the sampling and analysis process, chronologically, into discrete steps. Another standard procedure to know indoor air pollution in the polyurethane factories about isocyanates is biomonitoring using urine samples. Air sampling with biological sampling for isocyanates is the best procedure and perfect method for evaluation of isocyanate pollution in the polyurethane factories.

## CONCLUSION

The indoor air quality evaluation in polyurethane factories shows that increase of dry temperature in the workplaces caused increase of pollution. It may be due to physicochemical processes of isocyanates, which is thermopile. Similarly, the proportional humidity also increased the pollution in the workplace, which implies that humidity can condense isocyanate vapors in the workplaces. However, dimensions of the workplace have an opposite relation with pollution. Thus, pollution can further be reduced by enhanced rate of ventilation, air flow, and number of inlets and outlets that are responsible for dispersion of the pollutant concentration in the workplaces. The maintenance and correct use of injection sources and air-fed devices as well as training in work practices are other key elements of minimizing isocyanate exposure. Designing, installing, and starting of suitable ventilation system is most easy way of removing pollution in the work places. Besides that, use of personal protective equipment is also a must for workers inside the factories to avoid health hazards.
